# Technology of the photobiostimulation of the brain’s drainage system during sleep for improvement of learning and memory in male mice

**DOI:** 10.1364/BOE.505618

**Published:** 2023-12-05

**Authors:** Oxana Semyachkina-Glushkovskaya, Ivan Fedosov, Alexey Zaikin, Vasily Ageev, Egor Ilyukov, Dmitry Myagkov, Dmitry Tuktarov, Inna Blokhina, Alexander Shirokov, Andrey Terskov, Daria Zlatogorskaya, Viktoria Adushkina, Arina Evsukova, Alexander Dubrovsky, Maria Tsoy, Valeria Telnova, Maria Manzhaeva, Alexander Dmitrenko, Valeria Krupnova, Jürgen Kurths

**Affiliations:** 1 Department of Biology, Saratov State University, Astrakhanskaya Str. 83, 410012 Saratov, Russia; 2 Physics Department, Humboldt University, Newtonstrasse 15, 12489 Berlin, Germany; 3 Institute of Physics, Saratov State University Astrakhanskaya Str. 83, 410012 Saratov, Russia; 4 Department of Mathematics and Institute for Women’s Health, University College London, 25 Gordon Street, London, WC1H 0AY, UK; 5 Centre for Analysis of Complex Systems, Sechenov First Moscow State Medical University, Bolshaya Pirogovskaya 2, building 4, 119435 Moscow, Russia; 6 Institute for Cognitive Neuroscience, University Higher School of Economics, Moscow, Russia; 7 Britton Chance Center for Biomedical Photonics, Wuhan National Laboratory for Optoelectronics, Huazhong University of Science and Technology, Wuhan, China; 8 Institute of Biochemistry and Physiology of Plants and Microorganisms, Russian Academy of Sciences, Prospekt Entuziastov 13, Saratov 410049, Russia; 9 Potsdam Institute for Climate Impact Research, Telegrafenberg A31, 14473 Potsdam, Germany; 10 glushkovskaya@mail.ru; 11 alexey.zaikin@ucl.ac.uk

## Abstract

In this study on healthy male mice using confocal imaging of dye spreading in the brain and its further accumulation in the peripheral lymphatics, we demonstrate stronger effects of photobiomodulation (PBM) on the brain’s drainage system in sleeping vs. awake animals. Using the Pavlovian instrumental transfer probe and the 2-objects-location test, we found that the 10-day course of PBM during sleep vs. wakefulness promotes improved learning and spatial memory in mice. For the first time, we present the technology for PBM under electroencephalographic (EEG) control that incorporates modern state of the art facilities of optoelectronics and biopotential detection and that can be built of relatively cheap and commercially available components. These findings open a new niche in the development of smart technologies for phototherapy of brain diseases during sleep.

## 1. Introduction

The lymphatic drainage system plays a crucial role in protection us from infection and keeps a healthy balance of fluids throughout our body [1,2]. For a long time it was believed that there were no lymphatic vessels (LVs) in the central nervous system (CNS). Nevertheless, over two centuries, scientific evidence has accumulated providing the presence of drainage processes in the CNS [1,3]. It is still unknown whether LVs exist directly in the CNS [4,5]. However, the presence of LV in the brain’s meninges is widely accepted [6,7]. Despite the fact that the meninges do not belong to brain tissues, the role of meningeal lymphatic vessels (MLVs) in the removal of metabolites and toxins from brain tissues has been shown in many works [8–14]. Currently, MLVs is considered as a main part of brain’s drainage system (BDS) [3,15,16]. It is now known that dysfunction of MLVs plays an important role in the development of a large number of brain diseases, including Alzheimer’s (AD) [8,9,13,14] and Parkinson’s diseases [17], brain trauma [12] and tumors [18–20], intracranial hemorrhages [10,11]. Therefore, the development of methods to improve the functions of MLVs can contribute to progress in the treatment of these diseases.

Photobiomodulation (PBM) can be a promising technology for augmentation of functions of MLVs [13–16,21–29]. PBM is a therapeutic method based on the use of red or near-infrared light that has shown very promising results in the treatment of various types of dementia, depression and other neurocognitive disorders [30–38]. PBM has been recognized as safe by the U.S. Food and Drug Administration because PBM is a non-invasive approach without any side effects. Traditionally it was thought that the mechanisms of therapeutic effects of PBM are an increase in metabolism and microcirculation of brain tissue as well as reduction in oxidative stress and inflammation [36–40]. Recently, it was discovered that PBM can also effectively stimulate functions of MLVs [13–16,21–29]. Indeed, a series of experimental studies have shown that PBM stimulates the lymphatic excretion of toxins and metabolites from rodent brain tissues [13,14,22,23]. PBM activates lymphatic pumping via regulation of contraction cycle of LVs and phase of relaxation of LVs by activation of the intracellular reactive oxygen species and production of nitric oxide (NO) [23,25,26,41]. It is assumed that PBM can also lead to angio- and lymphangiogenesis, which might be another mechanism responsible for PBM-mediated stimulation of BDS [42].

Sleep is the time of natural activation of BDS [14–16,43–45]. During sleep the perivascular spaces (PVSs) increase facilitating the movement of brain fluids and thereby increasing the exchange processes between brain tissues and blood [43]. However, not all sleep is associated with activation of BDS. It is believed that only deep sleep or delta activity of the brain is accompanied by intense clearance of wastes, metabolites and toxins from the brain [43]. There is hypothesis that during deep sleep, the size of PVSs and the volume of interstitial fluids increase, promoting the removal of metabolites from brain tissue [15,16,43]. During wakefulness, the size of PVSs decreases and the exchange between fluids and brain tissue is suppressed. Thus, sleep is a driving factor for removal of unnecessary molecules from the brain.

Based on these new data, the idea of using PBM during deep sleep has been proposed as a promising new direction in the improvement of cognitive performance, executive functions, attention and memory [15,16,45–47]. However, PBM is typically used in the awake state [15,30–38]. There are no technologies for PBM during sleep [16].

Here, we propose the new technology for PBM under EEG control for stimulation of BDS during deep sleep and improvement of learning and memory in healthy male mice.

## 2. Methods

### 2.1. Subjects

Male C57BL/6 mice (25–28 g, 3 months age) were used in all experiments and were obtained from the National Laboratory Animal Resource Centre in Pushchino (Moscow area, Russia). The animals were housed under standard laboratory conditions with access to food and water ad libitum. All experimental procedures were performed in accordance with the “Guide for the Care and Use of Laboratory Animals”, Directive 2010/63/EU on the Protection of Animals Used for Scientific Purposes, and the guidelines from the Ministry of Science and High Education of the Russian Federation (№ 742 from 13.11.1984), which have been approved by the Bioethics Commission of the Saratov State University (Protocol No. 8, 18.04.2023). The mice were housed at 25 ± 2 °C, 55% humidity, and 12:12 h light–dark cycle (light: 08:00 am–08:00 pm). The mice adapted to the experimental conditions during one week before the beginning of the experiments to ensure acclimation to the housing room of the animal facility. The experiments were performed in the following groups: (1) no PBM, EEG control of wakefulness; (2) no PBM, EEG control of deep sleep; (3) PBM during deep sleep; (4) PBM during wakefulness; n = 7-8 in each group in all sessions of the experiments.

### 2.2. Technology of PBM under EEG control

A two-channel cortical EEG was recorded. The mice were implanted with two silver electrodes (tip diameter: 2–3 µm) located at a depth of 150 µm in coordinates (L: 2.0 mm and P: 2 mm) from the bregma on either side of the midline under inhalation anesthesia with 1% isoflurane (Sigma-Aldrich, St Luis, USA, at rate 1 L/min N_2_O/O_2_—70/30 ratio). The head plate was mounted and small burr holes were drilled. Afterward, EEG wire leads were inserted into the burr holes on one side of the midline between the skull and the underlying dura. EEG leads were secured with dental acrylic (Zhermack SpA, Badia Polesine, Venice, Italia). Ibuprofen (Bhavishya Pharmaceuticals Pvt. Ltd., Hyderabad, Telangana, India, 15 mg/kg) for the relief of postoperative pain was provided in their water supply for 2 to 3 days prior to surgery and for 3 days post-surgery. The mice were allowed 10 days to recover from surgery prior to beginning the experiment.

**Fig. 1.**
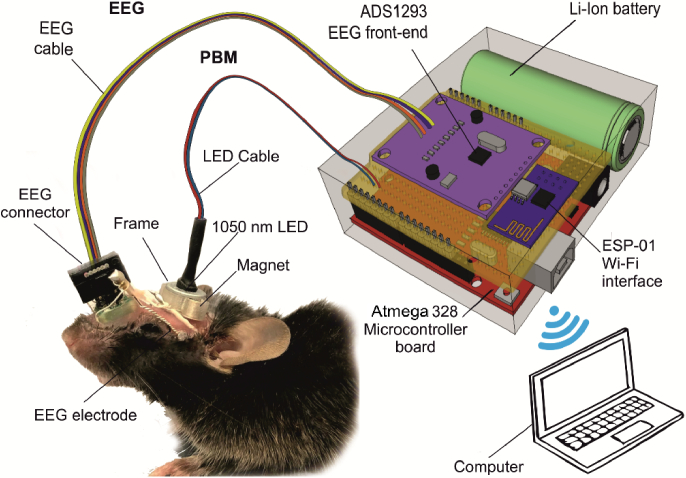
Technology of PBM under EEG control in unanesthetized mice.

EEG recording was performed with a tethered EEG system using ADS1293 (Texas instruments, Dallas, USA) Low-Power, 3-Channel, 24-Bit analog front-end for biopotential measurements. Initialization and data transfer were performed with Atmega328 (Atmel, San Jose, California, USA) microcontroller via SPI interface. The same microcontroller was used to detect non-rapid eye movement (NREM) sleep in real time. The instrument was powered with 18650 Li-ion battery. ESP-01 (Espressif Systems, PRC) module was used for Wi-Fi communication between the instrument and standalone PC. The instrument was placed on top of the homecage and connected to the mouse with 0.3 m long flexible 4 wire cable attached to a head mounted miniature connector soldered to 4 screw electrodes. The connector allows for easy plug of EEG instrument to mouse without anesthesia (Fig. 1). Note that anesthesia strongly affects the brains’ functions and BDS which makes is necessary to avoid the use of anesthesia in our experiments [48].

**Fig. 2.**
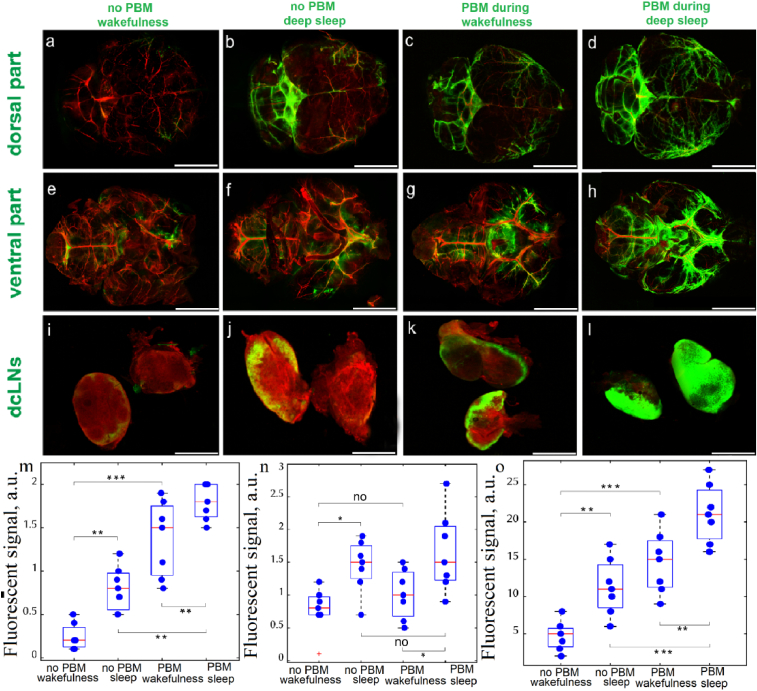
The confocal imaging of changes of BDS during wakefulness and sleep without and after single PBM treatment in healthy mice: a-d – Representative images of FITCD spreading in dorsal part of the mouse brain in wakefulness (a and c) and deep sleep (b and d) without PBM (a and b) and after PBM (c and d); e-h – Representative images of FITCD spreading in ventral part of the mouse brain in wakefulness (a and g) and deep sleep (f and h) without PBM (e and f) and after PBM (g and h); i-l -Representative images of FITCD accumulation in dcLNs in wakefulness (i and k) and deep sleep (j and l) without PBM (i and j) and after PBM (k and l). Scale bar for a-h – 3000 µm, for i-l – 1000 µm; m-o - Quantitative analysis of the fluorescent signal from FITCD (a.u.) in dorsal (m) and ventral (n) parts of brain as well as in dcLN (o), n = 7 in each group; * - p < 0.05, ** - p < 0.01, *** - p < 0.001, the ANOVA test with post hoc Duncan test.

A 1050 nm LED with output power of 50 mW in 2853 SMD housing was used for PBM. The LED driver was controlled with pulse width modulation (PWM) output of the EEG instrument microcontroller. The LED was connected to the instrument with 0.3 m long 2 wire flexible cable and mounted into miniature 3D printed frame with a pair of cylindrical magnets 3 mm in diameter and of 3 mm height each. That allows for easy attachment of the LED frame to M3 steel washer glued at mouse skull surface. LED operates in PWM mode at 1 kHz modulation frequency. The washer hole of 3.5 mm in diameter acts as an aperture limiting PBM area to 0.1 cm^2^ at the skull surface.

LED output power of optical radiation is 50 mW and the area limited with a washer is 0.1cm^2^. Thus at continuous operation mode power density is 500 mW/cm^2^ that complies with ANSI Z136 for maximal permissible exposure (MPE) of skin with 1050 nm NIR radiation. When operating in PWM regime at 1 kHz with duty less than 10% the instant and averaged power densities both at least 10 time less than MPE. Duty of 2% corresponds to 10 mW/cm^2^. For 17 min (1020s) long procedure at 2% PWM duty the LED delivers 10 J/cm^2^ dose over irradiated skull surface.

The instrument was controlled with a PC with software developed using LabVIEW (National instruments, Texas, USA). The software enables EEG recording, monitoring of the instrument operation and remote configuration of NREM sleep detection and PBM procedure. The EEG signal was digitized at 2.4 kSa/s, and filtered with a fifth-order digital Butterworth bandpass filter with the lower cutoff frequency of 0.5 Hz and the upper one of 250 Hz. For each 1 second long record Fast Fourier transformation was used to estimate power of EEG signal within frequency bands delta (0.5 Hz - 3.5 Hz), theta (4 Hz - 7.5 Hz), alpha (8 Hz - 12.5 Hz) and beta (13 Hz - 35 Hz), denoted as *P*_Δ_, *P*_Θ_, *P*_A_ и *P*_B_, respectively together with the integral spectral power *P*_Σ_ of the signal within the band 0.5 - 35 Hz. 15 minutes after EEG and LED were connected to the mouse, the peak value of *P*_Σ_ in wake conditions was measured and stored as a threshold value for sleep detector *P_w_*. The 1 s record is scored as NREM sleep if the logic value of *NREM* = (*P*_Δ_ > *P*_A_) & (*P*_Δ_ > *P*_B_) & (*P*_Θ_ > *P*_A_) & (*P*_Θ_ > *P*_B_) & (*P*_Δ_ > (*P*_A_ + *P*_B_)) & (*P*_Δ_ > *P*_Θ_) &(*P_w_ <* *P*_Σ_) is “true”. When 20% of 30 s epoch (6 of 30 consequent 1s records) were scored as NREM [49] then NREM sleep stage of animal was detected, and PBM LED is automatically turned on to run for 17 minutes at given PBM. Once configured the instrument was operated autonomously, logging its operations to be monitored via Wi-Fi connection. Wakefulness, NREM and rapid eye movement (REM) sleep were defined as described in our previous studies, where we demonstrate the EEG patterns and spectrum characteristics of wakefulness, NREM and REM sleep in mice [14,42]. Sleep scoring in mice has been extensively discussed in literature. Representative spectra of mice EEG for NREM, REM and wake states can be found in [50–52]. Slow wave activity within delta frequency band 0.5 ¬– 3.5 Hz is the key for NREM sleep scoring.

### 2.3. Behavioral tests

#### 2.3.1. Pavlovian-instrumental transfer (PIT)

**Fig. 3.**
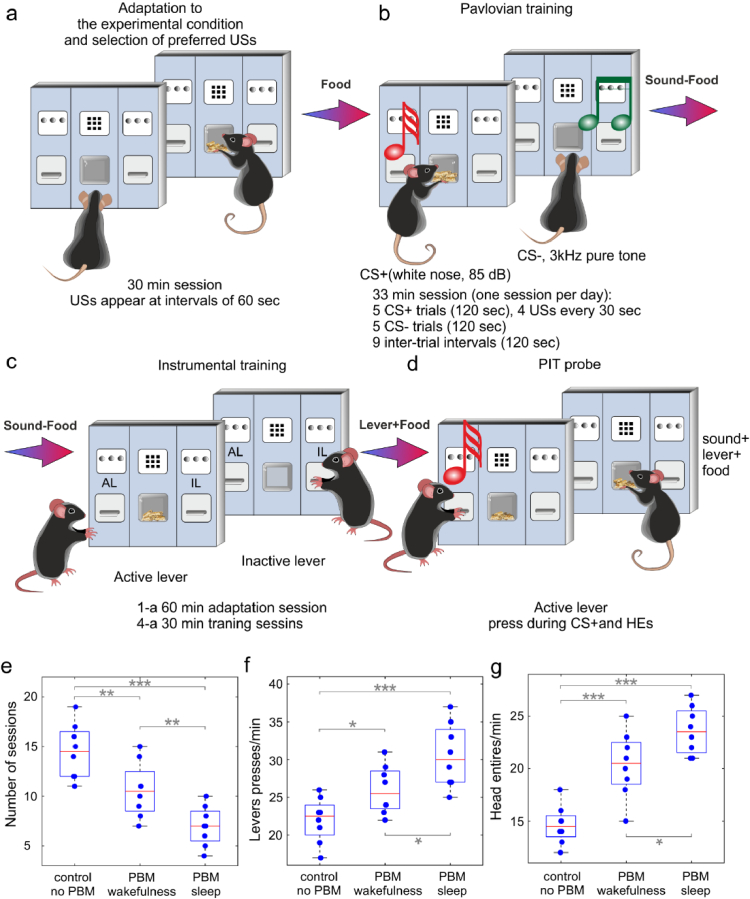
Pavlovian-instrumental transfer for the study of effects of PBM course during sleep and wakefulness on learning in health mice: a – d – The performance of PIT, including adaptation to the experimental environment and choice of preferred USs (a), Pavlovian training to discriminate sound (b) and active lever (c) associated with a reward, the PIT probe (d); e-g - Quantitative analysis of learning in the tested groups, including evaluation of session’s numbers that were required to reach PAVD (e), the number of AL lever’s pressing during CS+, and head entries into the receptacle; n = 8 in each group, * - p < 0.05, ** - p < 0.01, *** - p < 0.001, the ANOVA test with post hoc Duncan test

Here we used the method published in Ref. 53. The test was conducted in operant chamber (25 × 20 × 15 cm) housed within sound (85 dB white noise and 3 kHz pure tone) and light (infrared LED located inside the receptacle to detect head entries (HEs) into the receptacle). A pellet dispenser for delivery of 12-mg reward pellets (seeds of sunflowers, Grums, Minsk, Belarus) was located inside of the receptacle in the center of chamber. Ultrasensitive response levers were 7 cm apart on both sides of the magazine. Speakers were positioned 7 cm above the levers. The test started with the 30 min sessions during first 3 days to select a group of mice that prefer the same food and to adapt these mice to the experimental condition when food pellets (unconditioned stimulus, USs) appeared in the magazine with a random interval (RI) during 60 sec (Fig. 3(a)). Afterward, mice underwent daily the Pavlovian discrimination training sessions (38 min duration, one session per day) to differentiate one auditory food-attached conditioned stimulus (CS+, 85 dB white noise) from other auditory stimulus (CS−, 3 kHz pure tone) with the absence of reward (Fig. 3(b)). Each session includes the five CS + (120 min)/CS−(120 min) trials with a random order and an inter-trial interval (ITI, 120 sec) between CS + and CS−. Within each CS + session, 4 USs were delivered every 30 sec. The criterion for the Pavlovian discrimination (PAVD) were > 200 HEs/session (during 5 CS + trials) in the receptacle, where HSs should be during the first 15 sec of CS + trial, i.e. when no food was yet presented.

In the next step, mice were trained to press one of 2 levers to receive USs (Fig. 3(c)). The pressing the active lever (AL) was accompanied by a reward in the form of USs and pressing the inactive lever (IL) was not encouraged by USs. Left and right levers were at the same distance (5 cm) from the receptacle. Session were performed daily and lasted during 60 min (adaptation) and 30 min (progress in training). The instrumental training success along 4 increasing intervals between pressing AL and the US delivery was assessed according to the following criteria: (1) >20 pressing the active lever during a 60-min adaptation session; (2) >150 pressing AL when USs delivered every 15 sec after pressing AL during a 30-min training session; (3) >300 pressing AL when USs delivered every 30 sec after pressing AL during a 30-min training session; (4) >400 pressing AL when USs delivered every 60 sec after pressing AL during a 30-min training session.

After instrumental training, a Pavlovian-instrumental transfer (PIT) test was conducted and repeated three times every next day. The duration of PIT was 43 min with a 5-min start and 41-min session followed by 38-min session, including the five CS + (120 min)/CS−(120 min) trials with a random order and a ITI-120 sec between CS + and CS−. The successful responses were evaluated CS+, CS − and ITI separately and calculated the number of AL presses and HEs.

#### 2.3.2. Object-location memory task

**Fig. 4.**
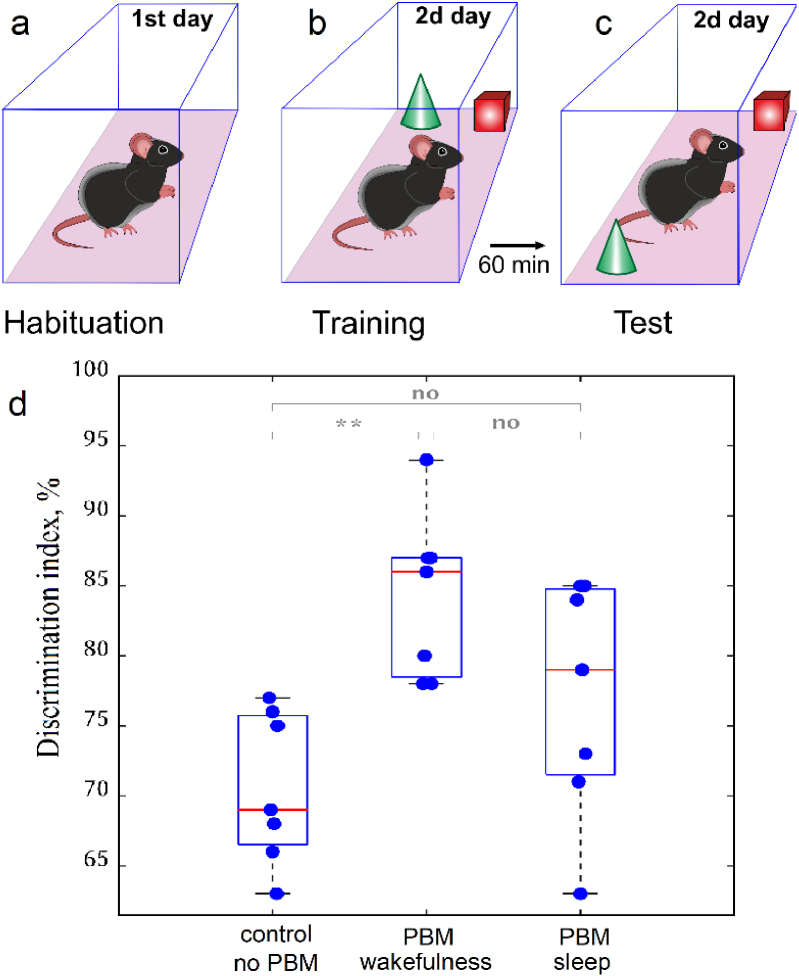
The study of effects of PBM during sleep and wakefulness on spatial memory in healthy male mice: a – Adaptation of mice to the experimental environment; b – Exploration 2 objects the same materials but different forms; c – Differentiation of new location of one object (green cone), while other (red square) remains on the same position; d - Quantitative analysis of performance of the 2-objects-location memory test in the tested groups, n = 7 in each group, ** - p < 0.01, the ANOVA test with post hoc Duncan test.

This test is based on the tendency of mice to spend more time exploring a novel object than a familiar one, including situation when an object changes its location. We used the 2-object-location memory location test described in Ref. 54. In the first trial (habituation phase), mouse is allowed to explore freely the empty box [70 cm (L) × 70 cm (W) × 30 cm (H)] during 5 min (Fig. 4(a)). In the next day, during second trial (training phase), mouse was places on the same box having two identical objects (red square and green cone with the same size, structure and material) on one place in the box opposite to each other and allowing mouse to freely explore the objects for 5 min (Fig. 4(b)). These colors were chosen because mice can only distinguish between blue and green. The weight of the objects was the same and sufficient so that the mice could not move them. Following an interval of 60 min, the third testing trial was performed for 5 min in the same day. Mouse was placed in the same box with one of the objects remaining in the same location as in trial 2 and the second object moved to a new location (Fig. 4(c)). A mouse is considered to be exploring an object when its nose is within 2 cm of the object. The third trial was continuously recorded using camera. The time for exploring the familiar object (T1), the time for exploring the object changed location (T2) and the total time for exploring both familiar and to-be-changed location objects were measured. Furthermore, the object-location discrimination index was calculated by using the formula (T2/(T1 + T2))x100 that was expressed in the percentages.

### 2.4. PBM for BDS during sleep and wakefulness

To study the PBM effects on BDS, we analyzed dye spreading in the whole brain after single PBM application using confocal microscopy. An amount of 5 µL of fluorescein Isothiocyanate (FITC)-dextran (FITCD, Sigma, St Louis, United States) was injected into the right lateral ventricle (AP - 1.0 mm; ML - 1.4 mm; DV - 3.5 mm) at a rate of 0.1 µL/min using microinjector (Stoelting, St. Luis, USA) with a Hamilton syringe with a 29-G needle (Hamilton Bonaduz AG, Switzerland). The implantation of chronical polyethylene catheter (PE-10, 0.28 mm ID x 0.61 mm OD, Scientific Commodities Inc., Lake Havasu City, Arizona, USA) into the right lateral ventricle was preformed according to the protocol reported by Devos et al. [55].

The head plate with LED was fixed in the region of the parietal and interparietal bones using dental acrylic (Zhermack SpA, Badia Polesine, Italia) under inhalation anesthesia with 1% isoflurane (Sigma-Aldrich, St Luis, USA, at rate 1 L/min N_2_O/O_2_—70/30 ratio). The LED was fixed to the head plate with two screws and was places on the parietal region, where the largest number of MLVs is located [7] (Fig. 1). The LED was performed during 61 min using following sequences: 17 min – LED, 5 min – pause, 17 min – LED, 5 min – pause, 17 min – LED as we have determined by a random selection in our previous studies [13,14,22,23,25,28,29]. The irradiance at skull surface does not exceed 0.5 W/cm_2_. Dose for PBM during single PBM treatment was 30 J/cm^2^ and for 10 days PBM course it was 0.3 kJ/cm^2^.

To study the PBM effects during wakefulness and deep sleep on BDS, the intracerebroventricular injection of FITCD was performed at 08:00 am under the EEG monitoring of wakefulness and after 08:00 pm at the time of EEG monitoring of NREM during 3h-observation. The time of 8 am and 8 pm for the intracerebroventricular injection of FITCD was chosen due to the light regime of vivarium and to standardize the protocol to start the experiment at the time of the natural transition to sleep or awakening. To keep the same time for the distribution of FITCD in the waking and sleeping states, mice that did not show NREM during 3h-observation were not included in the studies. The *ex vivo* optical study of FITCD distribution in the brain fluid system was performed 3 h after the intracerebroventricular injection of FITCD. Afterward, the whole brain imaging from dorsal and ventral aspects as well as the deep cervical lymph nodes (dcLNs) was performed using confocal microscopy.

The 10-days-PBM course during sleep or wakefulness was performed daily when mice underwent to PIT and the 2-obects-location memory test. The PBM under ECG monitoring was performed during observation of NREM (synchronized activity with high amplitude, which is dominated by low-frequency delta waves (0–4 Hz) comprising >30% of EEG waveforms/epoch) or wakefulness (low-amplitude and high-frequency dynamics >10%, 8–12 Hz).

The imaging was performed using Nikon A1R MP upright confocal microscope (Nikon Corp., Tokyo, Japan) with CFI Plan Apochromat Lambda D 2X (Nikon Corp., Tokyo, Japan) installed in a focusing nosepiece. Brain samples were submerged in a buffer solution in a Petri dish placed on the microscope stage. The top surface of each sample was covered with a 25 mm × 25 mm × 0.17 mm glass cover slide. Each brain image was captured as a stack of 5 stitched large images over 4 × 4 fields of view each. Image resolution was 3636 × 3636 pixels at 6.11 µm/pixel. Z step was 222 um. The resulting image was obtained as maximum intensity Z projection of all 5 images of the stack. The method enables to obtain extended along 1 mm depth of focus of high resolution image. The confocal images were captured in two channels: 488 nm excitation/525 emission was used to image FITCD distribution; and 640 nm excitation/700 nm emission for Evans Blue dye. dcLNs were imaged identically but only single field of view was captured for each stack. Image processing was performed using Fiji open-source image processing package [56]. Image processing procedures were identical for each pair of images (control and laser-treated samples) for each channel to ensure an accurate comparison of the fluorescence intensity.

### 2.5. Statistical analysis

All statistical analysis was performed using Microsoft Office Excel and SPSS 17.0 for Windows software. The results were reported as a mean value ± standard error of the mean (SEM). The inter-groups differences in all series experiments were evaluated using the ANOVA test with post hoc Duncan test. The significance levels were set at p < 0.05 for all analyses. No statistical methods were used to predetermine the sample size.

## 3. Results

### 3.1. PBM during sleep vs. wakefulness better stimulates BDS in healthy mice

In the first step, we analyzed the changes of BDS during wakefulness and sleep without and after single PBM treatment of mice (Fig. 2).

Our findings revealed that NREM was accompanied by stronger spreading of FITCD in both dorsal and ventral aspects of the brain as well as by its accumulation in dcLNs compared with wakefulness (0.80 ± 0.04 vs. 0.24 ± 0.02, p = 0,001399 for dorsal part of the brain; 1.44 ± 0.06 vs. 0.77 ± 0.05, p = 0,033662 for ventral part of the brain; 11.29 ± 0.55 vs. 4.71 ± 0.28, p = 0,002428 for dcLNs). The PBM during wakefulness and especially during sleep caused an increase in the FITCD distribution in the brain and its removal from CNS to dcLNs compared with mice without PBM (sleep + PMB vs. sleep, not PBM: 1.80 ± 0.03 vs. 0.80 ± 0.04, p = 0,000065 for dorsal part of the brain; 1.66 ± 0.09 vs. 1.44 ± 0.06, p = 0,373858 for ventral part of the brain; 21.14 ± 0.57 vs. 11.29 ± 0.55, p = 0,000105 for dcLNs; wakefulness + PBM vs wakefulness, no PBM: 1.37 ± 0.06 vs. 0.24 ± 0.02, p = 0,000065 for dorsal part of the brain; 1.01 ± 0.05 vs. 0.77 ± 0.05, p = 0,584763 for ventral part of the brain; 14.57 ± 0.60 vs. 4.71 ± 0.28, p = 0,000105 for dcLNs). Thus, PBM during sleep stimulated BDS higher than during wakefulness (1.80 ± 0.03 vs. 1.37 ± 0.06, p = 0,009807 for dorsal part of the brain; 1.66 ± 0.09 vs. 1.01 ± 0.05, p = 0,015901 for ventral part of the brain; 21.14 ± 0.57 vs. 14.57 ± 0.60, p = 0,002428 for dcLNs).

### 3.2. PBM during sleep vs. wakefulness better improves learning and memory in healthy mice

Next, we analyzed the PBM effects during sleep and wakefulness on learning and memory in healthy mice. To study learning in mice, we used PIT, when mice were trained to associate a sound with the delivery of food (Fig. 3(b)). Later, mice were exposed an instrumental training where they learned to press a lever to get food without the sound (Fig. 3(c)). Finally, mice were presented again with the opportunity to press the lever, this time both in the presence and absence of the sound (Fig. 3(d)). The results of reward-seeking response showed that trained mice pressed the lever more in the presence of the sound than without, even if the sound has not been previously paired with lever pressing. Thus, sound-food association transferred to the instrumental situation which indicated successful leaning.

Our results show that all mice (100%, n = 8) from the control group took 15 sessions to reach criterion for PAVD (Fig. 3(e)). The both courses of PBM during sleep and wakefulness improved learning in mice which all (100%, n = 8 in each group) could get criterion for PAVD for 7 (p = 0,000077) and 11 (0,006293) sessions, respectively. Thus, PBM facilitates learning. However, the effect of PBM on cognitive functions were better in sleeping vs. awake mice (p = 0,009736). On the PIT test, mice from all tested groups pressed on AL vs. IL significantly more often during CS + than during CS − or ITI (Fig. 3(f)). In addition, in all mice HEs were also more often during CS + than during CS − or ITI (Fig. 3(g)). However, mice from the control group pressed on AL during CS + lesser than mice from the PBM + sleep and PBM + wakefulness groups. Thus, mice without PBM made more mistakes during CS + than mice received PBM (p = 0,000185 between sleep + PBM and sleep, no PBM; p = 0,033729 between wakefulness + PBM and wakefulness, no PBM), i.e. the PBM course increased the number of correct responses pressing on AL during CS + (Fig. 3(f)). However, after the PBM course during sleep the corrected responses were more often than after the PBM during wakefulness (p = 0,01847) (Fig. 3(g)). Finally, HEs were increased by PBM vs. the control group, especially by the PBM during sleep vs. wakefulness (p = 0,000070 between sleep + PBM and sleep, no PBM; p = 0,000268 wakefulness + PBM and wakefulness, no PBM; p = 0,015562 between PBM during sleep and wakefulness) (Fig. 3(g)).

Next, we analyzed the effects of PBM course on spatial memory, including cognition and discrimination using the 2-object-location memory test. This test is based on the property of mice to learn the novel location of object spending more time for this processes vs. for a familiar ones. Test occurred in the open box, to which mice were first habituated for 5 min. In the next day, two objects of similar material but different forms (red square and green cone) were presented for 5 min on one position in front of each other. In 60 min, the similar objects were presented again but green cone was changed place, while red square remained position.

Our findings revealed that in all tested groups mice spent more time for exploring of to-be-changed object vs. familiar (Fig. 4). The course of PBM during sleep but not during wakefulness were accompanied by an increase in the discrimination index compared with the control suggesting that PBM only during sleep contributes to a better memorization of space, location and movement of objects in it (p = 0,002117 between sleep + PBM and sleep, no PBM; p = 0,140801 between wakefulness + PBM and wakefulness, no PBM). Thus, using the 2-object-location memory test, we estimated that PBM during sleep vs. wakefulness better improves memory in healthy mice.

## 4. Discussion

In this study on healthy male mice, we clearly demonstrate the effectiveness of PBM for stimulation of BDS that was associated with PBM-mediated improvement of learning and memory that was more pronounced in sleeping vs. awake animals. The mechanism by which PBM affects BDS has not been sufficiently explored. There are series of experimental data showing that PBM can stimulate the drainage and cleaning functions of the lymphatic vessels [13–16,21–29]. Li et al. found that PBM causes an increase of production of nitric oxide (NO) in the lymphatic endothelial cells (LECs) contributing contractility and pumping of LVs [41]. Indeed, NO plays an important role in regulation of lymphatic vascular wall tone [57,58]. The contraction cycle of LVs is regulated by mechanical and electrophysiological mechanisms. The relaxation phase is accompanied by a locally generation of NO in LECs in response to transiently elevated shear forces. The NO production in LECs causes vasodilation via stimulation of soluble guanylate cyclase that converts guanosine triphosphate to cyclic guanosine monophosphate, which activates protein kinase G. These changes cause the opening of calcium (Ca^2+^)-activated potassium channels and its reuptake. The reducing of the Ca^2+^ intracellular level prevents phosphorylation of myosin light-chain kinase leading to vasodilation after contraction. NO-induced vasorelaxation allows for diastolic filling of LVs and thus prepares them for their next contraction [59–61]. Thus, PBM can stimulate BDS by regulation of lymphatic pumping via the PBM-mediated effects on the NO generation in LECs and NO-modulation of phases of relaxation and contractility of the lymphatic vasculature.

In our recent paper, we clearly demonstrated that PBM-mediated increase in NO production in LECs that is associated with dilation of MLVs but without any changes in the cerebral blood flow [41]. This may be due to the fact that the effects of low intensity PBM are realized mainly superficially in the meninges, where MLVs and large cerebral sinuses are located. Therefore, we can see response to PBM from MLVs but not from the cerebral blood vessels, which are localized deeper in the cortex. Since we use low level of PBM, it can be not enough to induce response from the large cerebral veins (the sagittal and transverse sinuses).

How PBM can improve cognitive function remains poorly understood. Emerging evidence indicates the important roles of BDS in the resistant to neurocognitive disorders [8,9,14,63–68]. Indeed, dysfunction of MLVs results in less drainage of brain fluids to the cervical lymph nodes [8,11,12,19,28,41,42]. Such disruption of MLVs also results in cognitive impairment and behavioral alterations [8]. Increasing lymphangiogenesis of MLVs via administration of the vascular endothelial growth factor improves the drainage of macromolecules to the cervical lymph nodes of elderly mice [18,20,62–67]. Finally, disruption of MLVs worsens mouse models of AD [8]. Collectively, these findings suggest that dysfunction of BDS might provide an important contribution to age-related cognitive decline and neuro-degenerative disease [62–64].

In this study, we used LED 1050 nm for PBM-mediated stimulation of MVLs during sleep, while in our previous investigations we used laser 1267 nm for PBM [13,14,22–29]. The biological effect of both 1267 nm and 1050 nm optical radiations is related with direct singlet oxygen excitation. Wavelength of 1267 nm pumps a ground state oxygen molecule into the first excited singlet state, with no additional vibrational energy. The band is relatively broad ±20 nm. The band 1065 nm involves a transition of a ground state molecule into the first excited singlet state, with concurrent excitation of its first vibrational level. The band is also relatively broad ±15 nm. The absorption coefficient is smaller than the 1270 nm transition. After excitation, the vibrational energy decays very fast releasing heat to the environment, so for any practical biological purpose excitation at 1065 nm is equal to excite at 1270 nm. Using 1065 nm is preferable in biological research because of the tenfold reduction in water absorption as compared to 1270 nm [68]. The rationale of our research was to replace the expensive laser light source with cheap and reliable commercially available LEDs with emission band centered at 1050 nm and FWHM bandwidth of 40 nm. Although the emission band of LED partially only partially overlapped with singled oxygen band, we had demonstrated the significant effect of PBM using LED light source that can be very useful for the application of PBM technique to humans. Our result corresponds to the common trend of the use of LEDs instead of lasers for PBM [69].

We assume that the mechanisms of PBM for BDS and brain functions might be different in sleeping and awake subjects, which requires the detailed research [16,45–47]. Further animal and clinical studies of better therapeutic effects of PBM during sleep vs. wakefulness will shed light on optimization of PBM for successful phototherapy of neurocognitive diseases, including light wavelength, power intensity, treatment duration, and comfort light application on the head in horizontal position.

Typically, in experimental and clinical studies, PBM is performed in awake subjects [15,30–38]. In our recent review, we suggested that there are no commercial devices for simultaneous PBM and sleep monitoring, which significantly limits the clinical investigations the therapeutic effects of PBM during sleep [16]. We discussed that the development of sleep-PBM technologies is urgently needed and could become a breakthrough step in the progress of AD therapy in humans [15,16]. The technology proposed in this study opens new perspectives of development of promising PBM devices for night phototherapy of brain diseases and its implementation in clinical practice. The PBM can be also combined with near infrared spectroscopy and laser speckle contrast imaging techniques for advanced assessment of PBM effect on the brain function [70–75].

## 5. Conclusion

In sum, in the first time, we clearly demonstrate simple and reliable technique for PBM during deep sleep. We present the new technology of PBM under EEG-control that incorporates modern state of the art facilities of optoelectronics and biopotential detection and that can be built of relatively cheap and commercially available components. The overall cost of the homemade instrument do not exceed $200 and thus the parallel PBM of 10 or more animal can be performed in parallel using e.g. 10 of such setups. Our results clearly demonstrate that PBM can be a promising method for augmentation of BDS thereby contributing restoration of brain functions, including learning and memory. However, our data show that PBM during sleep vs. wakefulness better stimulates BDS, promoting better improvement of learning and spatial memory. These findings shed light on our knowledge about the nature of PBM and open a new niche for sleep-therapy of various brain diseases, including neurocognitive disorders.

## Data Availability

The data that support the findings of this study are available on request from the corresponding author.
